# Complete Genome Sequence of a Virulent Newcastle Disease Virus Strain Isolated from a Clinically Healthy Duck (*Anas platyrhynchos domesticus*) in Pakistan

**DOI:** 10.1128/genomeA.00730-16

**Published:** 2016-07-28

**Authors:** Abdul Wajid, Shafqat F. Rehmani, Muhammad Wasim, Asma Basharat, Tasra Bibi, Saima Arif, Kiril M. Dimitrov, Claudio L. Afonso

**Affiliations:** a Quality Operations Laboratory, University of Veterinary and Animal Sciences Lahore, Lahore, Pakistan; b Institute of Biochemistry and Biotechnology, University of Veterinary and Animal Sciences Lahore, Lahore, Pakistan; c Exotic and Emerging Avian Viral Disease Research Unit, Southeast Poultry Research Laboratory, U.S. National Poultry Research Center, ARS, USDA, Athens, Georgia, USA

## Abstract

Here, we report the complete genome sequence of a virulent Newcastle disease virus (vNDV) strain, duck/Pakistan/Lahore/AW-123/2015, isolated from apparently healthy laying ducks (*Anas platyrhynchos domesticus*) from the province of Punjab, Pakistan. The virus has a genome length of 15,192 nucleotides and is classified as member of subgenotype VIIi, class II.

## GENOME ANNOUNCEMENT

Newcastle disease (ND) is a highly contagious viral disease in birds caused by virulent strains of avian paramyxovirus serotype 1 (APMV-1), also known as Newcastle disease virus (NDV) (1). NDV has a single-stranded negative-sense RNA genome with six transcriptional units (3ˊ-NP-P-M-F-HN-L-5ˊ). Multiple avirulent and virulent ND viruses have been isolated from domestic and wild bird species, and wild waterfowl are considered to be natural reservoirs of NDVs of low virulence (2). Although there are some exceptions (3, 4), ducks generally show no clinical signs of ND when infected with highly virulent NDV (5).

As a result of an ND surveillance program in different avian species in Pakistan, we isolated virulent NDV strains classified as members of a recently identified subgenotype, VIIi, circulating in poultry and pet birds in Pakistan (6–8). These viruses have already spread through Asia, the Middle East, and East Europe, causing outbreaks of Newcastle disease with significant illness and high mortality in poultry, suggesting the existence of a fifth panzootic (1, 6). Here, we report the lack of significant genetic changes in the complete genomes of viruses previously reported in chickens that apparently spilled over into ducks. Swab samples collected from apparently healthy domestic ducks reared inside a poultry farm were inoculated in 9-to-11-day-old embryonating chicken eggs (NDV-specific-antibody free). A hemagglutinating sample was confirmed as APMV-1 by a hemagglutination inhibition (HI) assay (9). Viral RNA was extracted from the allantoic fluid using TRIzol LS, per the manufacturer’s recommendations (Invitrogen, USA). Reverse transcription was performed using random hexamers and RevertAid Premium reverse transcriptase (RT), according to the manufacturer’s protocol (Thermo Scientific, USA). The complete genome was sequenced as described previously (8), and the BioEdit software was used for sequence assembly and editing (10).

The complete genome length of the isolated virus (designated duck/Pakistan/Lahore/AW-123/2015) was 15,192 nucleotides. The sequence analysis of duck/Pakistan/Lahore/AW-123/2015 revealed polybasic amino acid residues between positions 113 and 116 of the fusion protein cleavage sites and a phenylalanine at position 117 (^113^RRQKR↓F^117^). Such an amino acid motif of the fusion protein cleavage site is considered typical for virulent NDV (9). Phylogenetic and comparative analysis revealed high genetic identity (99.11 and 99.11%) to recently isolated and characterized subgenotype VIIi strains from chickens in Pakistan (GenBank accession numbers KM670337 and KP776462, respectively), and 99.18% to a strain from Indonesia (GenBank accession no. HQ697254). The detection of subgenotype VIIi viruses in ducks indicates the possibility of transmission of the virus into waterfowl. The current scenario highlights the importance of a vigilant surveillance program in this region where ND is endemic.

### Nucleotide sequence accession number.

The complete genome sequence of NDV strain duck/Pakistan/Lahore/AW-123/2015 has been deposited in GenBank under the accession no. KU845252.
